# Training medical students in health promotion: twenty years of experience at the Faculty of Medicine of the University of Geneva

**DOI:** 10.15171/hpp.2017.42

**Published:** 2017-09-26

**Authors:** Thomas Mattig, Philippe Chastonay, Emmanuel Kabengele, Laurent Bernheim

**Affiliations:** ^1^Institute of Global Health, Faculty of Medicine, University of Geneva, Geneva, Switzerland; ^2^Health Promotion Switzerland, Bern, Switzerland; ^3^University of Fribourg, Fribourg, Germany; ^4^Vice-Dean’s Office for Medical Education, Faculty of Medicine, University of Geneva, Geneva, Switzerland

**Keywords:** Health promotion, Medical education, Curriculum reform

## Abstract

**Background:** In most cases, the work of medical doctors, be they general practitioners or specialists, involves some dimension of health promotion (HP). There is thus ample justification for increasing the awareness of medical students vis-à-vis HP and its relevance for their future practice.

**Methods:** In the context of a major curriculum reform (problem-based learning [PBL]) at the Faculty of Medicine of the University of Geneva in the mid-1990s, several steps were taken to strengthen HP throughout the curriculum and include HP in its key domains as defined by the Ottawa Charter (OC).

**Results:** First, the *political dimension of HP* was developed in a series of first- and fifth-year lectures and third-year workshops; second, community action was strengthened through a third-year one-month community immersion program; third, the development of personal skills was integrated into second- and third-year PBL cases and into fourth-and fifth-year learning activities in clinical settings as well as second- and third-year HP electives; in terms of reorienting health services, the chosen approach included the development of a HP-specific track in the context of a Certificate of Advanced Studies (CAS) in Community Health and a Master of Advanced Studies(MAS) in Public Health. Furthermore, *a supportive intra-university environment* was created through a collaborative convention with Health Promotion Switzerland, which is in charge of coordinating HP in Switzerland.

**Conclusion:** In our view, HP teaching for medical students seems all the more relevant given that future medical doctors will have to take care of an increasing number of patients likely to develop chronic non-communicable diseases.

## Introduction

In most cases, the work of medical doctors, be they general practitioners or specialists, involves some dimension of health promotion (HP). There is thus ample justification for increasing the awareness of medical students vis-à-vis HP and its relevance for their future practice.^1^

Marc Lalonde, the Canadian Minister of National Health and Welfare in the 1970s, first shaped the concept of HP in his report on the health of Canadians.^2^ Ultimately, his reflections were key to the development of the Ottawa Charter (OC) in 1986 and its main approaches, i.e., “advocacy, empowerment and mediation” in 5 action areas, namely building healthy public policy, creating supportive environments, strengthening community action, developing personal skills and reorienting health services.^3^ In 2003 the General Medical Council’s *Tomorrow’s Doctors* (second edition) further stressed the importance of exposing medical students to HP topics.^4^

In the context of a major curriculum reform (problem-based learning [PBL]) at the Faculty of Medicine of the University of Geneva in the mid-1990s,^5^ several steps were taken to strengthen community health^6^ and HP throughout the curriculum.

Our article presents the HP training activities progressively implemented at the Faculty of Medicine of the University of Geneva over a 20-year period.

## Materials and Methods

Considering HP in its key action areas as defined by the OC, the Faculty progressively implemented a series of HP learning activities ranging from the first year of medical studies to the fifth (the sixth being a clinical clerkship rotation year); furthermore, a Certificate of Advanced Studies (CAS) in Community Health^7^ and a Master of Advanced Studies (MAS) in Public Health^8^ were modified, i.e., for each a specific HP track was elaborated in collaboration with Health Promotion Switzerland, which is in charge of coordinating HP programs at the national level.

## Results

Keys to the successful implementation of HP teaching activities were the following elements:

- It was part of a major curriculum reform with a switch from frontal lectures to PBL during the preclinical years and more bedside teaching during the clinical years.
- It benefited from the fact that in the context of the curriculum reform, the public health dimension was considered as deserving of more consideration. This was a critical point, and the support from the various curriculum coordination committees was crucial.
- Its development was facilitated by the introduction of elective courses according to the recommendations of the Bologna Process.
- Its introduction and strengthening took place progressively over a period of several years.
- It has received support from Health Promotion Switzerland, which has mobilized teachers and has made available its network of contacts (field professionals).

The developed and implemented HP training activities can be summarized as follows:

*• OC action area – Building healthy public policy*

The political dimension of HP was developed in a series of first- and fifth-year lectures and third-year workshops focusing on the socio-economic role of the physician and on the organization of the health system. The topics discussed are summarized in Table 1.

*• OC action area – Strengthening community action*

Community action was strengthened through a third-year one-month community immersion program where small groups of students had to investigate the bio-medico-social and economic dimensions of a given health problem in the community by meeting and interviewing patients and families affected by the problem, health professionals in charge of the problem (general practitioners [GPs], medical specialists, public health officers, health economists, nurses, social workers, etc) as well as political authorities, researchers in the specific field and representatives of non-governmental organizations (NGO) active in the field. At the end of the month, each group had to present their work in a report, an oral presentation in front of their peers and a poster that summed up the network involved with the investigated topic.^9^ Examples of investigated topics include *Breast cancer screening program at state level: challenges and opportunities; Preventive measures in high-risk pregnancies; Vaccination against common childhood infectious diseases: what can the GP do to convince opposing parents?; HP activities targeting the elderly; Melanoma prevention strategies targeting young people; Palliative care versus euthanasia: a complementary approach to end-of-life?; Health-promoting activities in general practice; Bike and health.* Box 1 illustrates the cumulated perception the students had of the program over the years collected in a SWOT (strengths, weaknesses, opportunities, threats) grid.^10^

*• OC action area – Developing personal skills*

The development of personal skills was integrated into second-and third-year PBL cases and into fourth- and fifth-year learning activities in clinical settings as well as second- and third-year HP electives. Table 2 shows some integrated disease-prevention and health-promotion topics discussed in the basic-science and clinical-science PBL modules. During the HP electives, which represent 10% of the total ECTS points and were taken by roughly 10% of the students, students were put into active learning situations and assigned to elaborating HP projects. Developed projects targeting University of Geneva students included *Reduction of stress; Promotion of physical activities; Promotion of tools to enhance good sleep.* Evaluations of the electives program by the students showed high satisfaction, continued interest and strong commitment.

*• OC action area –Reorienting health services*

Given the academic setting, the chosen approach for reorienting health services included the development of a HP-specific track in the context of a CAS in Community Health and a MAS in Public Health. Since the 2 programs are heavily community and project centered, the objective was to initiate the planning and implementation of community HP programs and HP research projects. Examples of such projects appear in Table 3.

*• OC action area – Creating a supportive environment*

Furthermore, a supportive intra-university environment was created through a collaborative convention with Health Promotion Switzerland, which is in charge of coordinating HP in Switzerland. The collaboration allowed mobilizing the Foundation’s HP experts as teachers, tutors and project supervisors. It also ensured funding for HP activities at the Faculty of Medicine and provided the basis for joint research activities related to the federal non-communicable diseases strategy.^11^

In the 20 years since the curriculum reform, over 2500 students have been exposed to the various HP activities.

The difficulties encountered had various origins. Here are some of the most striking examples:

- Some tutors of the basic-science PBL modules were not comfortable with the integrated HP topics, which meant that these topics got less attention during PBL sessions and over the years were even in danger of disappearing.
- The organizational and logistical aspect of the community immersion activities proved to be quite time consuming, as did the tutoring of the students: this strained the resources of the teaching staff in charge of the program.
- So far, the HP electives failed to meet expectations with regard to students’ interest and participation: HP electives are in competition with many other courses, some of which are perceived by students as more important or more interesting in light of their future profession.
- The long-term upkeep of HP community projects initiated by the CAS and MAS students could frequently not been ensured.

## Discussion

In the aftermath of the OC, the World Conference on Medical Education in Edinburgh stated that medical education should “produce doctors who will promote the health of all people.”^12^ The drive for more HP education has been kept alive over the years, with, e.g., the report of a medical students conference in Bristol in 2006 proposing such HP learning objectives as being able “to promote the health of individuals and society” or being familiar with “the strategies of prevention.”^13^ The issue has further been raised/supported by political authorities stressing the importance of developing HP competencies among health care professionals, e.g., in Britain with the program *Putting Prevention First*^14^ or more recently in Switzerland with the program *Health2020.*^15^

The HP education activities implemented at the Faculty of Medicine of the University of Geneva go, in our view, in the direction of those recommendations.

We were faced with several challenges in implementing those HP teaching activities, even though we benefited from favorable conditions in the context of the curriculum reform. Indeed, the support of the various curriculum committees was key in implementing HP teaching activities. Furthermore, a close collaboration with the persons in charge of teaching public health and community medicine was crucial to accessing “teaching slots” and developing shared teaching activities. One of the major challenges we faced was the availability of competent staff in the field of HP to set up and carry out the program over the long term. Here we benefited from the support of Health Promotion Switzerland, which provided some of the HP expertise and teaching staff. Another challenge lay in identifying teaching methods attractive enough for students to engage in enthusiastically; the community immersion program was part of the solution in that it allowed students to explore any given health problem in its bio-psycho-social dimensions and the HP and disease prevention interventions likely to decrease the problem. A third challenge, as yet unresolved, was to ensure the long-term upkeep of HP community projects initiated by the CAS and MAS students. In many cases to date, long-term funding could not be obtained, which brought the projects to an early end.

Through the variety of educational approaches adopted (lectures, workshops, community investigations, integrated HP topics in basic-science and clinical-science PBL modules, project planning and implementation in the community), their distribution over the length of the curriculum and their adherence to the five OC action areas, the developed HP education activities respect, in our view, the recommendations of educational experts. Indeed, the community immersion program and the community project as implemented by the Geneva medical and public health students are coherent with Naidoo & Orme’s vision of an “expanding role for medical doctors in planning HP activities for local populations.”^16^ The integration of HP topics into clinical-science PBL modules responds to one of the challenges HP topics face in a medical curriculum, namely the clinical relevance, which would ultimately raise student interest as has been reported.^17,18^ The integration of HP topics into all the years of the curriculum, as was done in Geneva, is also recommended in the literature.^19^ Finally, the signing of a cooperation agreement between the University of Geneva and Health Promotion Switzerland allows us to envision further developments in HP education for medical students and health personnel in general.

Perhaps one setback that should be mentioned: the difficulty of developing HP activities in an integrative, multi-professional way, i.e., HP workshops and HP community projects that include students from medical schools, nursing schools, dietetics schools and physical therapy schools, a multidisciplinary and multi-professional approach as advocated by a large panel of experts.^20^ Each institution/track has its own catalogue of objectives and its own timeline, but each institution also has its prejudices regarding the others, which can be difficult to overcome.

## Conclusion

The various HP teaching activities developed at the Faculty of Medicine of the University of Geneva benefited from a specific dynamic related to a major curriculum reform. Their implementation was progressive and done in close collaboration with the persons in charge of public health and community medicine of the university. The support of Health Promotion Switzerland boosted the project and provided access to a HP-competent workforce.

In our view, HP teaching for medical students seems all the more relevant given that future medical doctors will have to take care of an increasing number of patients likely to develop chronic non-communicable diseases.

## Ethical approval

None to be declared.

## Competing interests

The authors declare that they have no competing interests.

## Authors’ contribution

PC and TM wrote the initial text. EK and LB reviewed and complemented it. All four have been key actors, at various levels, in the implementation of the different HP programs as described in the paper.

**Table 1:** Examples of topics taught/discussed (OC action area “Building healthy public policy”)

	**Topics**
Year 1	Social and environmental health determinants Health determinants in professional settings Determinants of disease chronicity Health promotion and disease prevention in general practice Motivational interviewing
Year 3	Organization of the health system and the role of various actors Regulations, incentives and constraints of medical practice Cost control of the health system: challenges and opportunities Economic evaluation of health care activities and public health measures Health care and disease prevention reimbursement mechanisms
Year 5	Public health screening strategies Health promotion and disease prevention strategies Occupational health issues and prevention measures Evidence-based health promotion and disease prevention

**Table 2:** Examples of integrated HP topics discussed in the basic-science and clinical-science PBL modules (OC action area “Developing personal skills”)

**Basic-Science PBL Modules**	**Examples (not exhaustive)**
Infectious Diseases	Vaccination strategies against influenza and poliomyelitis
Cellular Aging and Oncogenesis	Prevention strategies of colorectal carcinoma
Nutrition and Metabolism	Prevention of overweight/obesity among teenagers
Reproductive Health	Ethical issues related to assisted reproductive technology
Cardiovascular System	Prevention strategies of thromboembolic disease
Respiratory System	Respiratory allergy prevention measures
Excretion and Homeostasis	Hypertension
Infectious Diseases	Community prevention of sexually transmitted diseases
Immunity	Vaccination strategies
Neuroscience	Psycho-social consequences of psychiatric disorders
Musculoskeletal System	Prevention of falls/fractures among the elderly
**Clinical-Science PBL Modules**	
Introduction to Clinical Sciences	Health policies concerning the drug market
Surgery	Prevention of accident-related trauma
Gynecology–Obstetrics	Prevention of breast cancer; promotion of breastfeeding
Internal Medicine	Hepatitis B vaccination strategies
Community Medicine	Prevention of alcohol-related health problems
Psychiatry	Depression prevention strategies
Pediatrics	Health promotion measures in school settings
Neurology	Organization of long-term care of epileptic patients
Emergency Medicine	Medico-legal dimensions in emergency interventions

**Table 3:** Examples of implemented projects (OC action area “Reorienting health services”)

**Research projects**
Access to health services for the migrant populations in Switzerland: findings and limitations
Access to health care for victims of female genital mutilation in Switzerland
The challenges and expectations of relatives of people suffering from mental disorders
Medico-economic evaluation of maternal and perinatal care services of a new model of midwifery care
**Intervention projects**
“*Croque & bouge*” (chew& move): an obesity prevention program for young children
Development and implementation of cantonal tobacco prevention programs
Planning and implementing a sensory screening program in schools in a Swiss canton
Implementing a community health promotion project with nursing students

**Table:** 

**Box 1.** Cumulated perception (main points) the students had of the community immersion program over the years (OC action area “Strengthening community action”) collected in a SWOT (strengths, weaknesses, opportunities, threats) grid ***Strengths*** • Investigating a health problem in its various dimensions • Interacting with professionals from different backgrounds and with various community actors • Discovering the complexity of the disease experience of a patient and his/her family ***Weaknesses*** • Limited time for the investigation • Occasional difficulties in accessing health authorities and community actors ***Opportunities*** • Setting the foundation for a community health network • Contributing in some cases to the better community understanding of the investigated topic ***Threats*** • Superficiality due to limited time at disposal • Work overload

## References

[1] Wylie A, Davis AM. The rationale and historical context to justify the inclusion of health promotion in curricula. In: Wylie A, Holt T, eds. Health Promotion in Medical Education. From Rhetoric to Action. Oxford: Radcliffe Publishing; 2010. p. 1-2.

[2] Lalonde M (1974). A New Perspective on the Health of Canadians.

[3] WHO (1986). Ottawa Charter for Health Promotion.

[4] General Medical Council (2003). Tomorrow’s Doctors.

[5] Vu NV, Bader CR, Vassalli JD. The redesigned undergraduate medical curriculum at the University of Geneva. In: Scherpbier AJJA, van der Vleuten CPM, Rethans JJ, eds. Advances in Medical Education. Dordrecht: Kluwer Academic Publishers; 1997. p. 532-5.

[6] Chastonay P, Vu NV, Humair JP, Mpinga EK, Bernheim L (2012). Design, implementation and evaluation of a community health training program in an integrated problem-based medical curriculum: a fifteen-year experience at the University of Geneva Faculty of Medicine. Med Educ Online.

[7] Chastonay P, Zesiger V, Stoll B (2009). Enseignement de la santé publique, de la santé communautaire et des droits de l’homme à la Faculté de médecine de Genève : plus de 20 ans de partenariat avec les organisations internationales. Revue médicale suisse.

[8] Chastonay P, Jeannot E, Stoll B, Mattig T, Moretti R, Walker F (2017). A 25-year experience with a Project-centered Master in Public Health : key to public health relevance and educational efficacy?. Creat Educ.

[9] Chastonay P, Zesiger V, Klohn A, Soguel L, Mpinga EK, Vu N (2013). Development and evaluation of a community immersion program during preclinical medical studies: a 15-year experience at the University of Geneva Medical School. Adv Med Educ Pract.

[10] Sorensen L, Vidal RVV. Getting an overview with SWOT. Danish National Research Database. Technical University of Denmark; 1999. Available from: http://forskningsdatabasen.dk/catalog/2185776144. Accessed December 28, 2016.

[11] FOPH. National Strategy for the Prevention of Non-communicable Diseases. Bern: FOPH; 2016. Available from: https://www.bag.admin.ch/bag/fr/home/themen/strategien-politik/nationale-gesundheitsstrategien/strategie-nicht-uebertragbare-krankheiten.html. Accessed December 28, 2016.

[12] World Federation for Medical Education. The Edinburgh Declaration. Lancet. 1988;8068:464.

[13] Hilgers J, De Roos P (2007). European core curriculum--the students’ perspective, Bristol, UK, 10 July 2006. Med Teach.

[14] Department of Health. Putting Prevention First: Vascular checks – risk assessment and management. NHS; 2008. Available from: http://www.healthcheck.nhs.uk/document.php?o=227. Accessed December 28, 2016.

[15] Federal Council. Health2020. Bern; 2013. Available from: https://www.admin.ch/gov/fr/accueil/documentation/communiques.msg-id-47540.html. Accessed December 28, 2016.

[16] Naidoo J, Orme J (2000). Health promotion in the medical curriculum: enhancing its potential. Med Teach.

[17] Rego PM, Dick ML (2005). Teaching and learning population and preventive health: challenges for modern medical curricula. Med Educ.

[18] Bellas PA, Asch SM, Wilkes M (2000). What students bring to medical school: attitudes toward health promotion and prevention. Am J Prev Med.

[19] Gillam S, Maudsley G. Public Health Education for Medical Students: A Guide for Medical Schools. Cambridge: Department of Public Health and Primary Care, University of Cambridge; 2008.

[20] Frenk J, Chen L, Bhutta ZA, Cohen J, Crisp N, Evans T (2010). Health professionals for a new century: transforming education to strengthen health systems in an interdependent world. Lancet.

